# Abnormal auditory ERP N100 in children with dyslexia: comparison with their control siblings

**DOI:** 10.1186/1744-9081-5-26

**Published:** 2009-06-26

**Authors:** Charalabos Papageorgiou, Giorgos A Giannakakis, Konstantina S Nikita, Dimitris Anagnostopoulos, George N Papadimitriou, Andreas Rabavilas

**Affiliations:** 1 University of Athens, 1st Department of Psychiatry, Eginition Hospital, Athens, Greece; 2 National Technical University of Athens, School of Electrical and Computer Engineering, Biomedical Simulations and Imaging Laboratory, Athens, Greece; 3 University Mental Health Research Institute (UMHRI), Athens, Greece

## Abstract

**Background:**

Recent research has implicated deficits of the working memory (WM) and attention in dyslexia. The N100 component of event-related potentials (ERP) is thought to reflect attention and working memory operation. However, previous studies showed controversial results concerning the N100 in dyslexia. Variability in this issue may be the result of inappropriate match up of the control sample, which is usually based exclusively on age and gender.

**Methods:**

In order to address this question the present study aimed at investigating the auditory N100 component elicited during a WM test in 38 dyslexic children in comparison to those of 19 unaffected sibling controls. Both groups met the criteria of the International Classification of Diseases (ICD-10). ERP were evoked by two stimuli, a low (500 Hz) and a high (3000 Hz) frequency tone indicating forward and reverse digit span respectively.

**Results:**

As compared to their sibling controls, dyslexic children exhibited significantly reduced N100 amplitudes induced by both reverse and forward digit span at Fp1, F3, Fp2, Fz, C4, Cz and F4 and at Fp1, F3, C5, C3, Fz, F4, C6, P4 and Fp2 leads respectively. Memory performance of the dyslexics group was not significantly lower than that of the controls. However, enhanced memory performance in the control group is associated with increased N100 amplitude induced by high frequency stimuli at the C5, C3, C6 and P4 leads and increased N100 amplitude induced by low frequency stimuli at the P4 lead.

**Conclusion:**

The present findings are in support of the notion of weakened capture of auditory attention in dyslexia, allowing for a possible impairment in the dynamics that link attention with short memory, suggested by the anchoring-deficit hypothesis.

## Background

Dyslexia describes individuals having reading related difficulties despite normal intelligence, adequate training and satisfactory educational opportunities. Approximately 5–17% of the population is considered to have dyslexia [1,2]. Recent research has implicated deficits of the working memory (WM) operation and attention within the patterns of cognitive characteristics of dyslexia [3-5]. However, the underlying mechanisms and the cognitive characteristics of dyslexia still remain unclear [6-8].

Event-related potentials (ERP) provide an objective index of cognition-related brain activity because they reflect aspects of information processing that are relatively inaccessible using self-report or traditional behavioral techniques. The auditory N100 component of ERPs has been conceptualized as the physiological correlate of both attentional and working memory operation [9-13].

Previous studies involving dyslexic subjects in comparison to controls have investigated hypotheses relating information processing deficiency with patterns of N100 component using ERP methodology. The results of these studies have been variable. Several studies have found that subjects with dyslexia have reduced N100 amplitude [14-16] and prolonged latency [17] as compared to controls. It has been argued that this reduced and slowed activity within these early stages of information processing contributed to the language deficits of dyslexic children, [18,19]. On the other hand there are studies that failed to find a discrepancy between dyslexic children and controls [20] or even demonstrated opposite effects [21].

The reason for inconsistencies in the reported results may be the diversity in the study design and methodology. Variability is likely to arise at least partly from the fact that the normative data used possibly do not constitute a fair comparison for dyslexic subjects, because they are drawn from a control population selected to represent a not well-defined samples data except the matching of demographic data of age and sex. It is apparent, for example, that children of low socioeconomic status usually suffer more than do children of high socioeconomic status from problems that impair performance on cognitive tests. Therefore, a promising comparison might be an investigation between dyslexic subjects and their sibling controls, since both groups share similar family relations and socioeconomic status, thus eliminating most of the above confounding factors. At this point, it is worth noting that our team studying dyslexic children in comparison to their sibling controls found that dyslexic children manifested abnormal aspects of pre-attentive processing of information as they are reflected by P50 elicited during a working memory test [22].

In view of the above considerations, we hypothesized that the electrophysiological brain activity, as reflected by N100, in association with working memory operations, could be of significance in identifying possible pathophysiological mechanisms involved in dyslexia. Thus, the present study was designed to determine: (a) whether dyslexic children as compared to their sibling controls have similar or different features of N100 ERP component elicited during a working memory test; (b) whether dyslexic children have more difficulties than their normal siblings with regard to memory performance, and (c) the strength of association, if any, between memory performance and the amplitudes and latencies of the N100, and whether this association would be similar or different for dyslexic children and their sibling controls.

## Methods

### Subjects

Fifty seven (57) children participated in the study. Thirty eight (26 boys and 12 girls) of them were outpatient cases who had been diagnosed as having dyslexia according to the 10th edition of the International Classification of Diseases (ICD-10) and the rest 19 children (7 boys and 12 girls) were sibling controls of the dyslexic group. The selection of the group of the sibling controls was also based on the criteria of the 10th edition of the (ICD-10). The mean ages and the standard deviations for the dyslexic children and for the controls were 11.47 ± 2.12 (range 7–16 years-old) and 12.21 ± 2.25 years (range 8–16 years-old), respectively (non-significant age difference, t-test p = 0.232). These differences remain non-significant in comparisons between groups separately for boys and girls, as well as between genders separately for the controls and dyslexics. In each case, the following assessments were performed: child psychiatric examination, psychological examination and educational evaluation. The Wechsler Intelligence Scale for Children – Third Edition (WISC-III) [23] was used to obtain the IQ of each child. The assessment of educational attainment included reading, comprehension, spelling and arithmetic ability. A standard procedure was followed using a special test for the Greek language [24].

Participants did not enter the study if they had (a) clinically notable neurological disease (including seizure disorder), (b) a history of head injury, (c) hearing difficulties and (d) attention deficit disorder and hyperkinetic syndrome. Prior to participation in the examination, parents were informed about the aims of the study, received a full description of the procedure, and provided written consent. Children were tested individually. The investigators explained to each child the procedure and the children also gave their consent. The study was approved by the local ethical committee.

### Stimuli and procedure

**Figure 1:**
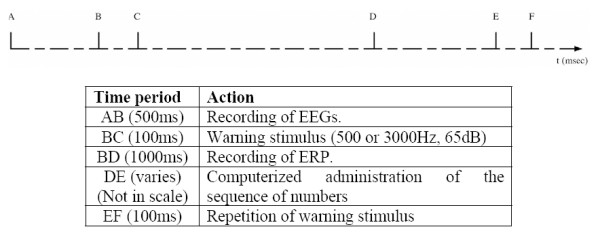
**Outline of the experimental procedure**.

The subjects were evaluated with the digit span Wechsler Auditory test [22,25]. For each trial of the experiment, rest EEG signal was recorded for 500 msec. A single sound tone of either high (3000 Hz) or low frequency (500 Hz) was presented to the subjects through earphones, followed by the numbers which had to be memorized. If the frequency of the signal tone was low the subjects had to recall the numbers in the same order as that presented, else (high frequency tone) the subjects had to recall the numbers in the reverse order. The total task consisted of 52 repetitions for a period of about 45 min. An outline of the procedure is provided in Figure 1.

**Figure 2:**
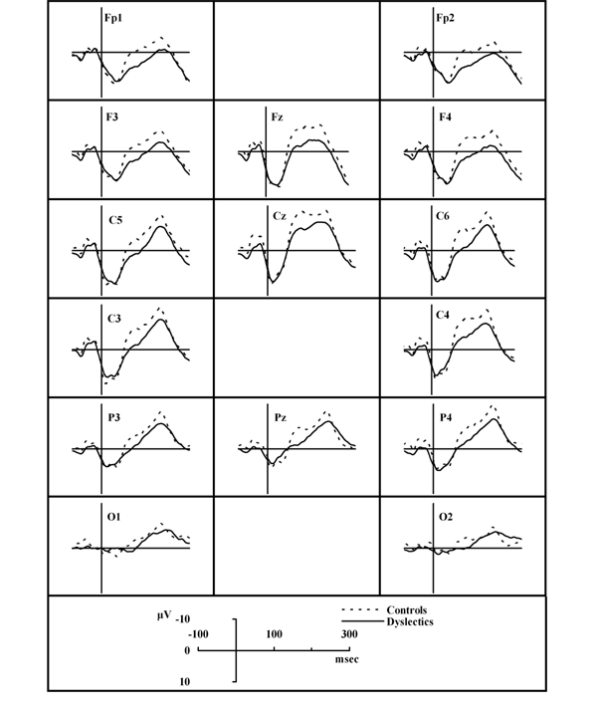
**Average ERP waveforms between dyslexic children and control group, induced by the high frequency tone**.

The children's EEG/ERP signals were recorded at 15 electrodes (Fp1, F3, C5, C3, Fp2, F4, C6, C4, O1, O2, P4, P3, Pz, Cz, Fz) according to the 10–20 international system, referred to both earlobes. Ag/AgCl electrodes were attached to the scalp with adhesive cream in order keep the electrode resistance below 5 kΩ. An electrode placed on the subject's forehead served as ground. The pass band of the amplifiers was set from 0.05 Hz to 35 Hz. During the recordings the subjects had their eyes closed in order to minimize eye movements and blinks. Eye movements were recorded through electro-oculogram (EOG) and recordings with EOG higher than 75 μV were rejected. Warning stimuli, as well as learning material (i.e. the numbers to recall), were presented binaurally via earphones at an intensity of 65 dB sound pressure level. The evoked biosignal was submitted to an analog-to-digital conversion, at a sampling rate of 1 kHz, and was averaged by a computerized system. An algorithm was used, which identified the N100 as the most negative peak in averaged lead curve, between 70 and 150 msec, after the warning stimulus. Peak amplitudes were measured relative to the mean amplitude of the 100-ms pre-stimulus baseline period and latency measurements were computed relative to stimulus onset (Figure 2).

As the N100 component is included in the array of early endogenous ERP components, which normally are modality specific, the ERP induced by the two modal stimuli were averaged separately [26]. In particular, two varieties of N100 waveforms were obtained, one (low N100) evoked by the low frequency modality (26 trials) for each lead in all participants and another (high N100) evoked by the high frequency modality (26 trials) for each lead in all participants.

The behavioral performance refers to the number of correctly recalled digits. The total digits presented in each session were 298, 149 digits for the part of the session which engaged the low frequency stimuli and likewise 149 for the part which engaged the high frequency stimuli.

### Statistical analysis

Preliminary analysis of the data showed that all the dependent variables did not deviate from the normal distribution. Furthermore, the amplitudes and the latencies were found to follow the multivariate normal distribution. This allowed for the application of parametric tests and specifically of multivariate parametric procedures.

The high and low frequency amplitudes and latencies of the N100 component, taken over the range of 70–150 msec, were separately subjected to multivariate analysis with age as covariate and group as the between-subjects factor, followed by multiple pairwise comparisons between the two groups with Bonferroni correction. Using age as a covariate controls for its effect on the ERP component. After finding the locations, at which there were statistically significant differences between the two groups, the leads were sorted in a descending order of the significance of the difference and stepdown procedures were applied. The purpose was to elucidate whether significant differences at more than one lead were due to high correlations between the lead amplitudes and/or latencies or were specific and independent for each lead. IQ scores were compared between the two groups with the t-test for independent samples. Finally, the association between the N100 amplitudes at the high and low frequency tone with memory performance for the two groups was analyzed using partial correlation, controlling for age. Differences between the correlation coefficients for the two groups were tested using the appropriate Fisher transformations. Statistical significance was set at the 0.05 level in all cases.

## Results

### Amplitudes and latencies

**Figure 3:**
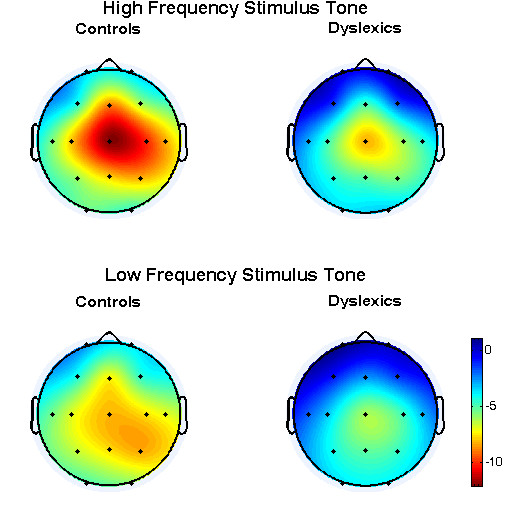
**Topological distribution of the mean amplitude values of the N100 component**.

No differences were observed for the mean latency values of the N100 component between the control and the dyslexics group, neither at the high nor at the low frequency tone. Conversely, as figure 3 shows, the mean absolute amplitudes of the N100 component for the control group was greater than for the dyslexics group for all leads, both at the high and at the low frequency tone. As post-hoc comparisons with Bonferroni correction showed, at the majority of the leads (7 out of 15 at high frequency, 9 out of 15 at low frequency), these differences achieved statistical significance. These results are shown in table 1 and table 2 respectively, where asterisks denote the leads, at which the mean absolute amplitude value for the control group is significantly greater than for the dyslexics group (one asterisk: p < 0.05, two asterisks: p < 0.01).

**Table 1:** Descriptive statistical measures of N100 ERP component amplitudes for high frequency stimulus type.

**High Frequency Stimulus Tone**					
**Electrode**	**Controls**		**Dyslexics**		**Post-hoc comparisons Sig. (2-tailed)**
		
	**Mean**	**SD**	**Mean**	**SD**	

**Fp1**	-3.44	4.45	-0.19	4.78	0.025*
**F3**	-4.31	4.07	-1.36	4.63	0.039*
**C5**	-5.55	5.76	-2.93	5.55	0.214
**C3**	-7.88	5.66	-4.72	5.58	0.103
**Fp2**	-3.37	3.34	0.46	5.11	0.009**
**F4**	-6.06	3.94	-1.73	5.80	0.008**
**C6**	-8.24	4.50	-4.53	6.35	0.056
**C4**	-9.96	4.30	-5.90	6.68	0.033*
**O1**	-5.35	5.33	-3.63	3.74	0.216
**O2**	-4.66	3.95	-3.31	3.69	0.289
**P4**	-8.21	4.73	-5.57	6.36	0.196
**P3**	-5.99	4.68	-4.41	4.58	0.408
**Pz**	-7.82	4.79	-5.24	4.87	0.103
**Cz**	-11.87	4.98	-8.17	5.48	0.026*
**Fz**	-8.76	4.30	-4.89	6.70	0.043*

**Table 2:** Descriptive statistical measures of N100 ERP component amplitudes for low frequency stimulus type.

**Low Frequency Stimulus Tone**					
**Electrode**	**Controls**		**Dyslexics**		**Post-hoc comparisons Sig. (2-tailed)**
		
	**Mean**	**SD**	**Mean**	**SD**	

**Fp1**	-2.55	4.20	1.06	4.13	0.007**
**F3**	-4.31	4.62	-0.87	3.37	0.005**
**C5**	-5.44	4.35	-1.92	5.13	0.031*
**C3**	-6.75	4.42	-3.25	5.41	0.040*
**Fp2**	-3.67	4.17	0.21	3.83	0.002**
**F4**	-4.58	3.96	-1.37	5.19	0.037*
**C6**	-6.87	5.07	-3.04	5.76	0.035*
**C4**	-7.48	5.31	-4.68	5.15	0.108
**O1**	-4.94	3.14	-3.67	4.29	0.412
**O2**	-5.78	3.02	-3.82	4.69	0.167
**P4**	-8.29	4.38	-4.69	5.37	0.025*
**P3**	-6.52	4.46	-4.24	4.23	0.116
**Pz**	-7.65	3.84	-5.22	4.11	0.065
**Cz**	-8.13	3.82	-6.05	5.80	0.189
**Fz**	-7.26	5.00	-2.58	6.36	0.013*

The dyslexics group can be divided into two subgroups: those children, whose siblings were also examined and dyslexics without normal siblings. Statistical analysis showed that these two subgroups were practically the same and differed in the same manner from the control group.

The N100 ERP component achieves its greater mean absolute amplitudes in frontal, central and parietal regions (Cz, C4, Fz, C6, P4, C3, Pz) for controls at both frequencies, in central, parietal regions (Cz, C4, P4, Pz, Fz, C3, C6) for dyslexics at high frequency and in central, parietal and occipital regions (Cz, Pz, P4, C4, P3, O2, O1) for dyslexics at low frequency stimulus type as it can be seen in Figure 3.

In both stimulus frequency types the lead with the maximal difference between the two groups was Fp2. Stepdown procedures proved that group differences found at the other leads could be attributed to the high covariance between the leads. This means that the leads at the N100 component act as an assembly, behaving in a coherent manner. Therefore dissimilar behaviour of the two groups is expected to be observed simultaneously over a large area of the scalp.

### Behavioral data

**Figure 4:**
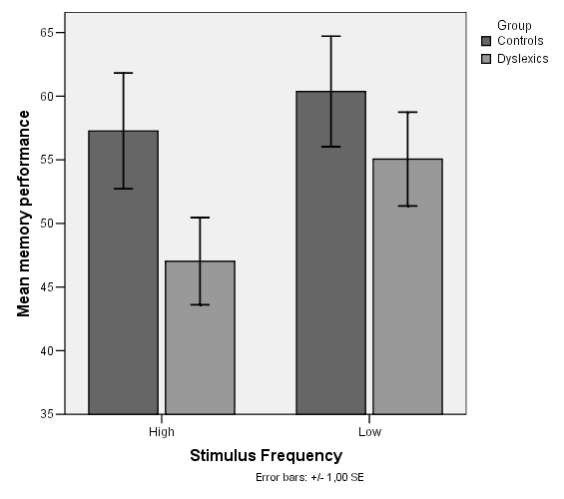
**Mean memory performance of controls and dyslexics for both stimulus type frequencies**.

No differences were observed between the compared groups with regard to IQ scores (dyslexics: 90.74 ± 11.86, controls: 93.60 ± 10.11, p-value: 0.420). Similarly, with regard to the memory performance, the main finding of the study is that dyslexic children did not differ in comparison to controls for both stimulus types. Better performance was observed for all subjects in forward digit span (low frequency stimulus type) than reverse. In high frequency (reverse digit span) where the mental task is more demanding, difference in memory performance between controls and dyslexics is more intense (t-test, p = 0.084) but it does not achieve statistical significance. These can be illustrated in figure 4.

### Correlations of N100 with memory

Table 3 shows the partial correlation coefficients for the two groups between N100 amplitudes at the high and low frequency tone with memory performance controlling for age.

**Table 3:** Partial correlation coefficients between N100 amplitudes and memory performance (*:p < 0.05, **:p < 0.001).

	**Frequency Stimulus Type**			
	
**Electrode**	**High**		**Low**	
	
	**Controls**	**Dyslexics**	**Controls**	**Dyslexics**
**Fp1**	-0.28	-0.01	-0.23	0.03
**F3**	-0.18	0.12	-0.25	0.24
**C5**	-0.54*	-0.06	-0.37	0.17
**C3**	-0.54*	0.05	-0.28	0.17
**Fp2**	-0.05	0.15	-0.07	0.16
**F4**	-0.25	0.11	0.01	0.01
**C6**	-0.49*	0.01	-0.37	0.07
**C4**	-0.35	-0.09	-0.11	0.20
**O1**	-0.15	-0.36*	-0.16	0.17
**O2**	-0.24	0.02	-0.08	-0.03
**P4**	-0.61**	0.02	-0.63**	-0.01
**P3**	-0.40	-0.02	-0.25	0.11
**Pz**	-0.20	-0.10	-0.21	0.01
**Cz**	-0.34	0.03	-0.01	0.01
**Fz**	-0.39	0.11	-0.08	0.07

Enhanced memory performance is related with increased N100 amplitudes (more negative value) for the control group and mainly in high frequency stimulus type. This relation is especially conspicuous for the amplitudes of the P4 electrode. Using the appropriate Fisher transformations, it was found that in high frequency stimulus type significant differences between the above correlation coefficients for the controls and dyslexics appear in electrodes C3, P4 and in low frequency stimulus type only in electrode P4.

## Discussion

The results showed that the dyslexic group demonstrated significantly reduced amplitudes of the N100 induced by the high frequency stimulus at Fp1, F3, Fp2, Fz, C4, Cz and F4 leads as well as significantly reduced amplitudes of the N100 induced by the low frequency stimulus at Fp1, F3, C5, C3, Fz, F4, C6, P4 and Fp2 leads. As far as memory performance is concerned, the main finding of the study is that dyslexic children did not fall short in a significant manner from their sibling controls,. However, enhanced memory performance in the control group is associated with increased N100 amplitude induced by high frequency stimuli at C5, C3, C6 and P4 leads and increased N100 amplitude induced by low frequency stimuli at P4 lead.

The patterns of the amplitudes of the N100 induced by both high and low frequency stimuli obtained in the present study suggest that children with dyslexia may demonstrate impairments in 'attentional operation' of information processing (as they are reflected by the N100 amplitudes elicited during a WM test) that involve or affect widespread brain areas. This suggestion seems to be in agreement with evidence elicited from recent research, demonstrating the implication of attentional alterations in the pathophysiology of dyslexia, as it is reflected by the N100 component [14,16,27].

In particular, Bonte and Blomert [16], investigating ERP correlates of implicit phonological processing during spoken word recognition in dyslexic and normally reading children (7–10 years), observed that the dyslexics manifested smaller N100 amplitudes at temporal electrodes than normal readers. These findings have been conceived of as indices of 'functional and/or anatomical anomalies in neural sources involved in speech processing in dyslexics'. Similarly, Pinkerton [14] found reduction of the N100 in a group of 14 boys with difficulties in reading, writing and spelling as compared to those of 18 matched controls (8–9 years). Interpreting these findings, the authors hypothesized that the reduced N100 in dyslexics might be understood as an index of impaired selective attention. A very recent study by Fosker and Thierry [27] investigated 12 dyslexic adults (mean age 20 ± 1 year) and 12 control adults (mean age 19 ± 1 year) using an oddball paradigm, reported that the dyslexic adults showed significantly smaller amplitudes of N100, concluding that these findings point to a deficit on the low-level perceptual processing capacity in association with attention. In corroboration to this idea seems reasonable to bring in mind that the N100 component can be elicited by 'pre-attentive' means [28].

Brunswick and Rippon [15] also found that normal controls exhibited significantly greater N100 amplitudes at the left temporal region during a dichotic listening test, while the dyslexic children exhibited approximately equivalent values of N100 amplitudes bilaterally. The authors regarded the divergence in N100 laterality as an indication of "inaccurate tuning of sensory information" leading to less reliable auditory representation.

Taken together – despite the use of divergent methodological approaches and eliciting procedures – these reports show that dyslexics demonstrate significantly smaller N100 amplitudes as compared to controls, hence indicating that dyslexia may be associated with abnormal processes mediating attentional operation. With regard to the locations that dyslexics demonstrate decreased N100 amplitudes, it is obvious that it concerns a widespread network within various brain regions.

As far as memory performance is concerned, the main finding of the study is that dyslexic children did not differ significantly in comparison to normal controls. This finding is in agreement with other related studies [29]. However a very large body of evidence points to the presence of a deficit in phonological short term memory in developmental dyslexia. [30-32]. Sources for divergent findings may be conceived in terms of both the heterogeneity of the study samples and differences in the measurement of the procedures. However, this still leaves open the possibility that genetic factors, may contribute to the lack of significant difference in memory performance observed in the present study. Given that developmental dyslexia is genetically associated [33,34] the possibility that the sibling controls of the current study were slightly dyslexic themselves cannot be completely ruled out. In this sense, the reports [35,36] provide additional explanatory hints underscoring the importance of the maturational age trajectory.

Finally, the observed association between the amplitude of the N100 and memory performance in the control group, in contrast to the lack of such an association in dyslexic children could be explained taking into account the anchoring-deficit hypothesis, which proposes that the deficit of dyslexics resides in the dynamics that link attention with short memory through the implicit formation of stimulus-specific anchors [32].

Certain limitations of this investigation warrant consideration. Firstly, the main findings need to be replicated in independent samples including unrelated controls and it is to be determined, whether there is an association in a task-specific manner or across tasks. Secondly, beyond of the advantage which incurs of the use of unaffected sibling controls it should be taken into account that possible confounding parameters might be implicated through this fact, such as the common genetic factors with their possible consequences. Hence future studies controlling for genetic factors as well as trait and state parameters in combination with more experiments that combine the time resolution of event-related potentials with the spatial resolution of brain imaging techniques may lead to clearer definitions of the brain function in relation to the current findings.

## Conclusion

The present findings provide evidence that dyslexic children may manifest impaired 'attentional operation' of information processing as reflected by N100 elicited during a working memory (WM) test. In addition these results acknowledge a possible impairment in the dynamics that link attention with short memory as suggested by the anchoring-deficit hypothesis.

## Competing interests

The authors declare that they have no competing interests.

## Authors' contributions

CP participated in the design of the study, designed the experimental procedure and the clinical protocol, participated in data acquisition, participated in analysis and interpretation of data and drafted the manuscript. GAG participated in the design of the study, participated in acquisition, analysis and interpretation of data and drafted the manuscript. KSN conceived and coordinated this study, participated in the interpretation of data and corrected the final version of this manuscript. DA participated and conceived in the design of the study, participated in the interpretation of data. GNP participated and conceived in the design of the study, participated in the interpretation of data. AR: conceived and coordinated this study and corrected the final version of this manuscript. All authors have read and approved the final manuscript.
